# Type A insulin resistance syndrome due to a novel heterozygous c.3486_3503del (p. Arg1163_Ala1168del) *INSR* gene mutation in an adolescent girl and her mother

**DOI:** 10.20945/2359-4292-2021-0305

**Published:** 2024-01-29

**Authors:** Serkan Bilge Koca, Melike Ataseven Kulali, Başak Güğüş, Hüseyin Demirbilek

**Affiliations:** 1 Afyonkarahisar Health Sciences University Faculty of Medicine Department of Pediatrics Afyonkarahisar Turkey Afyonkarahisar Health Sciences University, Faculty of Medicine, Department of Pediatrics, Division of Pediatric Endocrinology, Afyonkarahisar, Turkey; 2 Afyonkarahisar Health Sciences University Faculty of Medicine Department of Pediatrics Afyonkarahisar Turkey Afyonkarahisar Health Sciences University, Faculty of Medicine, Department of Pediatrics, Division of Pediatric Genetics, Afyonkarahisar, Turkey; 3 Afyonkarahisar Health Sciences University Faculty of Medicine Department of Medical Genetics Afyonkarahisar Turkey Afyonkarahisar Health Sciences University, Faculty of Medicine, Department of Medical Genetics, Afyonkarahisar, Turkey; 4 Hacettepe University Faculty of Medicine Department of Pediatrics Ankara Turkey Hacettepe University, Faculty of Medicine, Department of Pediatrics, Division of Pediatric Endocrinology, Ankara, Turkey

## Abstract

Mutations in the insulin receptor (*INSR*) gene may present with variable clinical phenotypes. We report herein a novel heterozygous *INSR* mutation in an adolescent girl with type A insulin resistance syndrome and her mother. The index case was a 12-year-old girl without obesity who presented with excessive hair growth, especially in the chest and back area, and hyperpigmentation on the back of the neck (acanthosis nigricans). Acanthosis nigricans was first observed at the age of 11 years. On physical examination, the patient had acanthosis nigricans and hypertrichosis with no acne. Systolic and diastolic blood pressure measurement was within the normal range for age and sex. Laboratory tests revealed fasting hyperglycemia, fasting and postprandial hyperinsulinemia, elevated HbA1c level, and biochemical hyperandrogenemia. Fasting plasma lipids were normal. A diagnosis of type A insulin resistance syndrome was considered, and *INSR* gene mutation analysis was performed. Next-generation sequence analysis was performed with the use of primers containing exon/exon-intron junctions in the *INSR* gene, and a novel heterozygous c.3486_3503delGAGAAACTGCATGGTCGC/p. Arg1163_Ala1168del change was detected in exon 19 of the *INSR* gene. In segregation analysis, the same variant was detected in the patient's mother, who had a milder clinical phenotype. We reported a novel, heterozygous, p. Arg1163_Ala1168del mutation in exon 19 of the *INSR* gene in a patient with type A insulin resistance syndrome, expanding the mutation database. The same mutation was associated with variable phenotypical severity in two subjects within the same family.

## INTRODUCTION

Insulin resistance is defined as the lack of insulin metabolic effects due to defects in the intracellular signaling pathways resulting from insulin receptor or post-receptor abnormalities. The main physical findings of insulin resistance are obesity, acanthosis nigricans, and hirsutism. Hyperglycemia, markedly increased insulin levels, elevated glycated hemoglobin (HbA1c) level, dyslipidemia, and hyperandrogenemia account for the biochemical features of insulin resistance (1). Although obesity is the most common accompanying condition, individuals with insulin receptor (*INSR*) gene mutations do not have features of metabolic syndrome (obesity, dyslipidemia).

The insulin receptor (encoded by the *INSR* gene) is a transmembrane heterotetrameric protein that belongs to the tyrosine kinase receptor family and consists of two alpha and two beta subunits. The alpha subunits are located outside the cell membrane and constitute the ligand-binding site, while the beta subunits – composed of extracellular, transmembrane, and intracytoplasmic domains – have tyrosine kinase activity. The *INSR* gene is located on chromosome 19, and each allele of this gene encodes for one alpha-beta half receptor. *INSR* gene mutations affecting one of these domains cause a wide range of clinical phenotypes, including the Donohue syndrome (leprechaunism), Rabson-Mendenhall syndrome, and type A insulin resistance syndrome. In the Donohue and Rabson-Mendenhall syndromes, patients may present in infancy or early childhood with severe symptoms of insulin resistance. In contrast, individuals with type A insulin resistance syndrome usually present with late-onset clinical findings of severe insulin resistance and hyperandrogenism. The insulin resistance is severe in individuals with biallelic (homozygous or compound heterozygous) *INSR* mutations and milder in those with heterozygous mutations (2–4).

We report herein the phenotypic characteristics of an adolescent girl who presented with type A insulin resistance syndrome in the peripubertal period and her mother who had a milder phenotype due to a novel heterozygous *INSR* gene mutation affecting the beta subunit of the insulin receptor.

## CASE PRESENTATION

**Figure 1:**
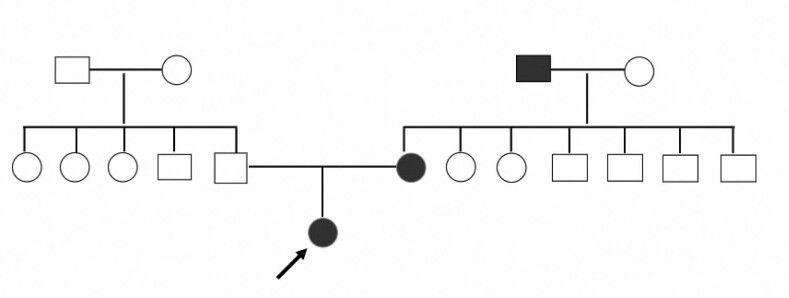
Pedigree of the family bearing the p. Arg1163_Ala1168del mutation. The arrow indicates the index case, and the black-filled boxes indicate individuals with diabetes mellitus.

A 12-year-old girl was seen at the pediatric endocrinology outpatient clinic with complaints of increased regional hair growth and hyperpigmentation of the skin (acanthosis nigricans), first observed at the age of 11 years and increasing over time. She was born to non-consanguineous parents via cesarean delivery at 36 weeks of an uneventful gestation. Her birth weight was 2,600 g (-1.5 standard deviation). Her family history revealed a mother diagnosed with polycystic ovary syndrome and type 2 diabetes mellitus. Her maternal grandfather also had type 2 diabetes mellitus (Figure 1). The mother was initially on oral antidiabetic drugs and required insulin treatment after the age of 30 years. The patient's family history was otherwise unremarkable.

**Figure 2:**
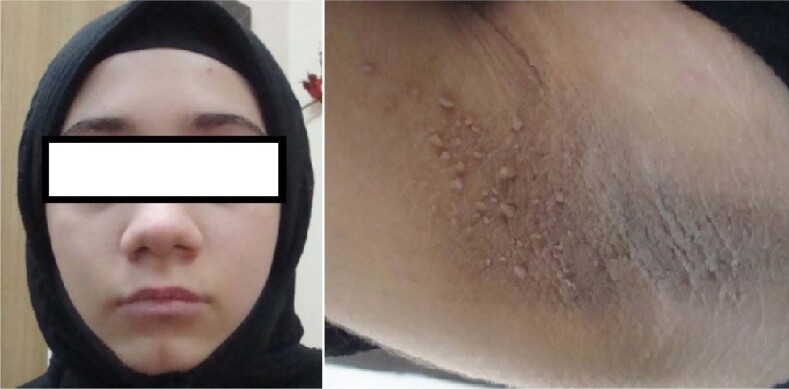
Patient's facial appearance and acanthosis nigricans (hyperkeratosis and mild papillomatosis with hyperpigmentation) in the axillary region.

At the first evaluation, the patient's height was 153.3 cm (55th percentile), weight was 52 kg (82nd percentile), and body mass index was 22 kg/m^2^ (86th percentile). On examination, her pubertal status was consistent with Tanner stage 4. She had hair growth on her back, arms, and legs, suggesting regional hypertrichosis, but had no acne. She also had severe acanthosis nigricans in her axilla, inguinal region, back of the neck, and antecubital region, which was first observed at the age of 11 years and had increased during the previous year (Figure 2). Her blood pressure was 110/70 mmHg. Spontaneous menarche had not occurred yet. On laboratory tests, she had fasting hyperglycemia, fasting and postprandial hyperinsulinemia, elevated HbA1c level, and biochemical hyperandrogenemia (Table 1). Fasting plasma levels of triglycerides, total cholesterol, and HDL cholesterol were normal. Luteinizing hormone level was high, and sex hormone binding globulin level was low. She had no imaging findings suggestive of hepatic steatosis or polycystic ovary syndrome on abdominal and pelvic ultrasonography examinations. A diagnosis of type A insulin resistance syndrome was considered, and *INSR* gene mutation analysis was performed.

**Table 1:** Clinical and laboratory characteristics of the patient at presentation and at 3 months of follow-up

	At presentation	At follow-up
BMI (SDS)	1.1	0.52
Acanthosis nigricans	Severe	Milder
Fasting glucose (mg/dL)	162	79
Fasting insulin (mIU/mL)	357	83
Fasting C-peptide (ng/mL)	19.1	5.7
HbA1c (%)	11	5.2
Glucose (postprandial, 2 hours) (mg/dL)	203	111
Insulin (postprandial, 2 hours) (mIU/mL)	663	>1,000
AST/ALT/GGT (U/L)	18/16/18	13/11/10
Triglycerides (mg/dL)	67	75
Total cholesterol (mg/dL)	131	141
HDL cholesterol (mg/dL)	60	64
LDL cholesterol (mg/dL)	80	71
FSH (mIU/mL)	7	6
LH (mIU/mL)	12	8
Estradiol (pg/mL)	44	45
Total testosterone (ng/dL)	98	108
DHEA-S (μg/dL)	192	331
17-hydroxyprogesterone (ng/mL)	3.2	1.1
Androstenedione (ng/mL)	6.7	5.6
SHBG (nmol/L)	9.8 (34-164)	9.8
Leptin (ng/mL)	15.3	N/A
TSH (μIU/mL)	2	N/A
Free T4 (ng/dL)	1.33	N/A
24-hour urinary microalbumin (mg/day)	9.1 (<30)	N/A

Abbreviations – ALT: alanine aminotransferase; AST: aspartate aminotransferase; BMI: body mass index; DHEA-S: dehydroepiandrosterone sulfate; HbA1c: glycated hemoglobin; GGT: gamma-glutamyltransferase; HDL: high-density lipoprotein; LDL: low-density lipoprotein; FSH: follicle-stimulating hormone; LH: luteinizing hormone; N/A: not available; SDS: standard deviation score; SHBG: sex hormone binding globulin.

Next-generation sequence analysis was performed with the use of primers containing exon/exon-intron junctions in the *INSR* gene, and a heterozygous c.3486_3503delGAGAAACTGCATGGTCGC/p. Arg1163_Ala1168del change was detected in exon 19 of the *INSR* gene. In segregation analysis, the same variant was detected in the patient's mother. This variant has not been previously reported in mutation databases (OMIM, NCBI dbSNP, ClinVar) or relevant literature (PubMed). The mutation was classified as “damaging” using *in silico* analysis tools (SIFT and PolyPhen) and evaluated as class 2 (“likely pathogenic”) according to the criteria set by the American College of Medical Genetics (ACMG) (5).

The patient's diabetes was managed with diet and metformin, which was started at a daily dose of 1,000 mg in two divided doses. A favorable glycemic control was achieved without insulin therapy. The HbA1c level decreased after the first 3 months of follow-up (Table 1). The phenotypic characteristics of the index case are shown in Figure 2.

Written informed consent was obtained from the patient and her mother.

## DISCUSSION

The present report described a novel heterozygous c.3486_3503del (p. Arg1163_Ala1168del) *INSR* mutation detected in an adolescent girl and her mother, with distinct clinical phenotypes suggesting variable phenotypical expression of heterozygous *INSR* mutations even in the same pedigree.

**Figure 3:**
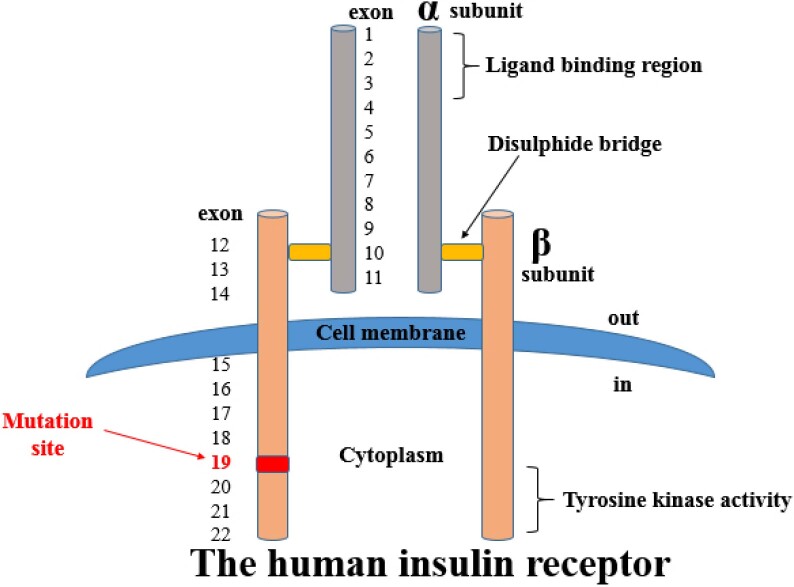
Schematic view of the human insulin receptor. The red arrow indicates the mutated region.

The heterozygous c.3486_3503del (p. Arg1163_Ala1168del) mutation lead to an 18 bp deletion in exon 19 of the *INSR* gene. This change occurred in the frame region, affecting the canonical sequence; it occurred 27 nucleotides downstream from the splice zone. According to the ACMG classification, this mutation is considered “likely pathogenic” because of a match with PM1 (UniProt protein *INSR*_HUMAN domain “protein kinase” has 25 non-VUS missense/in-frame/non-synonymous variants [20 pathogenic and 5 benign], pathogenicity = 80.0%, which is above the threshold of 50.0%), PM2 (variant not found in gnomAD exomes [good gnomAD exomes coverage = 94.7], variant not found in gnomAD genomes [good gnomAD genomes coverage = 30.1]), PM4 (protein coding length changes as a result of an in-frame variant in the *INSR* gene and is not in a repeat region), and PP3 (pathogenic computational verdict based on one pathogenic prediction from phyloP vs. no benign predictions) (5). *In silico* analysis suggested the variant to be pathogenic and predicted it to be the cause of the disease. A possible mechanism is that this variant could cause protein changes in the splice site, affecting the *INSR* signaling pathway, although additional protein analysis would be required for definitive results. The exon 19 region of the *INSR* gene encodes the beta subunit of the *INSR* protein and is known to have tyrosine kinase activity (Figure 3). Therefore, this amino acid change is predicted to affect the tyrosine kinase activity (thus, *INSR* signaling).

According to the 2021 ClinVar database, 551 different *INSR* gene variants have been described. Of these alterations, 177 are benign, 85 are likely benign, 205 are of uncertain significance, 10 are likely pathogenic, and 50 are pathogenic. Based on the molecular consequences of these gene variants, 6 are frameshift, 136 are missense, 10 are nonsense, 5 are splice-site, and 138 are untranslated region types. No database for these variants is available in Turkey, and the mutation detected in our patient is a deletion-type variant that has not been reported before in the literature. Notably, 42 of 551 *INSR* gene variants reported are deletion-type variants.

One heterozygous missense (p. Leu1150Pro) variant has been detected previously in the tyrosine kinase domain of *INSR* in a premenarcheal adolescent girl without obesity and with severe hirsutism, acanthosis nigricans, clitoral hypertrophy, deep voice, enlarged polycystic ovaries, severe hyperinsulinemia, and biochemical hyperandrogenism (6). The same mutation was also detected in the proband's mother and brother, who had a milder phenotype with undiagnosed insulin resistance. In addition, the proband had a heterozygous missense (p. Ala663Val) variant in exon 11 of the *SH2B1* gene that encodes for SH2B adapter protein-1. Of note, SH2B is an insulin-receptor adapter protein involved in the insulin signaling pathway. Therefore, the authors speculated that the *INSR* and *SH2B1* mutations were likely to affect insulin signaling synergistically and contribute to the more severe phenotype in the proband and the phenotypic variability within her family (6). In our study, no additional genetic studies were performed to exclude the possibility of a synergistic effect as a possible explanation for the phenotypic variation in our patient's family.

Clinicians categorize severe insulin resistance syndromes into two groups, *i.e.*, primary insulin pathway defects and secondary adipose tissue abnormalities. Primary insulin signaling defects, such as the *INSR* gene defect, are associated with marked hyperinsulinemia but not hyperlipidemia or fatty liver. In addition, elevated serum adiponectin, sex hormone binding globulin (SHBG), and insulin-like growth factor binding protein-1 (IGFBP-1) levels are detected. While dyslipidemia or fatty liver is observed in secondary fat tissue abnormalities (such as severe obesity), regional or generalized fat tissue loss can be detected in lipodystrophic syndromes (7). These clinical and laboratory findings can be helpful in detecting the specific etiology of severe insulin resistance and predicting its clinical course.

Type A insulin resistance is a rare clinical entity with an estimated incidence of 1/100,000, according to the U.S. National Library of Medicine Genetics Home Reference (8). The insulin receptor consists of two alpha subunits and two beta subunits. Alpha domains have ligand binding, and beta domains have tyrosine kinase activity. Therefore, mutations affecting the alpha subunit of *INSR* are suggested to cause more severe clinical findings (2,9,10). Elevated insulin causes hyperpigmentation and acanthosis nigricans in the skin by stimulating epidermal cell proliferation via hybrid insulin/insulin-like growth factor-1 (IGF-1) receptors. These mutations also cause clinical features of hyperandrogenism (including acne, hirsutism, and oligomenorrhea) by stimulating ovarian androgen synthesis. The cornerstone of management of insulin resistance syndrome due to *INSR* mutation consists of lifestyle modification, including regular exercise and dietary intervention. Treatment options include metformin monotherapy or combined with insulin for insulin resistance and diabetes mellitus, and combined oral contraceptives containing cyproterone acetate for hirsutism and menstrual disorders (11,12). In our patient, an initial dose of metformin 1,000 mg/day was effective without requiring strict dietary restrictions. The metformin dose was then reduced to 500 mg/day and later discontinued due to episodes of hypoglycemia. The HbA1c levels declined to 5.2% in the third month of dietary intervention and low-dose metformin treatment (Table 1). In adolescents, type A insulin resistance syndrome may be misdiagnosed as polycystic ovary syndrome (12). Indeed, in our proband's mother, who had the same mutation, the clinical manifestations first appeared in her 30s. She had developed hirsutism, diabetes, and fertility problems and was treated using various oral contraceptives and oral antidiabetic medications. Although achieving reasonable metabolic control in childhood and adolescence is possible, it has been reported that poor metabolic control and complications due to type 2 diabetes mellitus are more frequent in patients with type A insulin resistance syndrome in the long term (3,13).

The most frequent definition of severe insulin resistance by clinicians is the one associated with the insulin dose used by individuals with overt diabetes. However, as a clinical finding, acanthosis nigricans may be observed before hyperglycemia. With careful history taking, the manifestations of hypoglycemia – including the inability to fast for a long time or consumption of carbohydrates at frequent intervals – can be detected. Hyperglycemia associated with severe insulin resistance may occur secondary to postprandial hypoglycemia and go undetected for years (10). In some studies, severe postprandial hypoglycemia is also seen as a manifestation of defects related to primary insulin receptor or insulin signal transduction (14). Although the mechanisms of postprandial hypoglycemia are unclear, they are thought to be related to impaired hepatic insulin clearance due to primary insulin receptor defects or secondary hepatic steatosis (15). Some murine studies have shown that hyperinsulinemia associated with islet beta-cell hyperplasia can prevent hypoglycemia for years (16). Postprandial hypoglycemia occurring in the clinical course of insulin resistance or metformin treatment or both may have been effective in the hypoglycemia observed in our index case. For this reason, we recommend close monitoring of patients who are treated with an oral hypoglycemic agent or insulin.

Most mutations associated with type A insulin resistance identified to date have been described in case reports or series; therefore, genotype-phenotype types of studies are needed in Turkey and globally.

Family members with type A insulin resistance syndrome reported in the literature had features similar to those in our patient and her mother. The index case is generally a child, and the clinical manifestations (hirsutism, menstrual irregularities, polycystic ovary syndrome, acanthosis nigricans) occur more commonly during adolescence. Our patient's mother had an abnormal glucose tolerance test during pregnancy, hyperglycemia, hyperandrogenemia, and acanthosis nigricans that occurred at a later age. Patients diagnosed during childhood may have a history of being born small for gestational age, and episodes of fasting hypoglycemia can be a remarkable finding in some cases (17,18).

In conclusion, we reported herein a novel heterozygous mutation (p. Arg1163_Ala1168del) in exon 19 of the *INSR* gene in a patient with type A insulin resistance syndrome, a finding that expands the mutation database. The mutation was associated with variable phenotypical severity in two members of the same family. Monogenic insulin resistance syndrome should be considered in the differential diagnosis of individuals with acanthosis nigricans and hyperandrogenism without clinical features of metabolic syndrome like obesity or dyslipidemia.
